# High-dose statin pretreatment decreases periprocedural myocardial infarction and cardiovascular events in East Asian patients undergoing percutaneous coronary intervention: A meta-analysis of fifteen randomized controlled trials

**DOI:** 10.1097/MD.0000000000026278

**Published:** 2021-06-25

**Authors:** Jiahui Liu, Bin Zhang, Ming Chen, Bo Zheng

**Affiliations:** Department of Cardiology, Institute of Cardiovascular Disease, Peking University First Hospital, Beijing, China.

**Keywords:** Asian patients, high-dose statins, meta-analysis, periprocedural myocardial infarction

## Abstract

**Background:**

Numerous studies have shown that high-dose statin pretreatment may reduce the risk of periprocedural myocardial infarction (PMI) and short-term major adverse cardiac events (MACE) in western people undergoing percutaneous coronary intervention (PCI). However, the effects in East Asian patients are still controversial. The objective was to evaluate the effects of short-term high-dose statin (all types) pretreatment compared with the control (low-dose or no statin) on the reduction of the rate of MACE and PMI in East Asian patients.

**Methods:**

PubMed/Medline, EMBASE, and the Cochrane Central Register of Controlled Trials were systematically searched for randomized controlled trials (RCTs) in East Asian patients up to December 2019, in which short-term high-dose statin pretreatment was compared with control for patients undergoing PCI. The primary outcome measure was the incidence of MACE at 30 days. The secondary outcome measure was the incidence of PMI. The meta-analysis was performed with the fixed-effect model or random-effects model according to the heterogeneity. The meta-analysis was performed using RevMan 5.3 software (Cochrane Collaboration).

**Results:**

Fifteen RCTs that enrolled 4313 East Asian patients were identified. High-dose statin pretreatment was associated with a 54% relative reduction in 30-day MACE (OR, 0.46; 95% CI, 0.31–0.67; *P* < .001) and a 50% relative reduction in PMI (OR, 0.50; 95% CI, 0.34–0.76; *P* = .001).

**Conclusions:**

High-dose statin pretreatment can significantly reduce 30-day MACE and PMI for East Asian patients undergoing PCI.

## 1 Introduction

Coronary artery disease (CAD) is one of the most serious diseases that threaten human health, and it is associated with a heavy burden throughout the world. Cardiovascular diseases (CVDs) mostly comprise CAD, which is the leading cause of death in non-communicable diseases, and they accounted for approximately one-third of all deaths worldwide (17.9 million deaths) in 2012, as reported by the World Health Organization (WHO; http://www.who.int/gho/ncd/mortality_morbidity/en/). Percutaneous coronary intervention (PCI) is now the most common procedure that used in the invasive treatment of patients with CAD.^[1]^ Although the devices and surgical techniques have made remarkable improvements, there are still some serious complications that have not been eliminated during PCI. Periprocedural myocardial infarction (PMI) is a common complication, and it refers to PCI-related myocardial infarction, which has now been linked in large datasets to long-term adverse outcomes, including mortality.^[2]^ Pretreatment with statins was demonstrated to significantly reduce PMI and major adverse cardiac events (MACE) because of their anti-inflammatory effects.^[3,4]^ Several recent clinical trials have shown that high-dose statin therapy before the PCI procedure can significantly reduce the incidence of cardiovascular adverse events.^[5]^ However, the efficacy and safety of high-dose statins before PCI in East Asian patients are unclear because the evidence is limited to studies with low numbers of events and different clinical types of CAD. Thus, we systematically evaluated the clinical benefits of high-dose statin preloading before PCI in East Asian patients by conducting a systematic review and meta-analysis including all relevant randomized controlled trials (RCTs).

## 2 Methods

### 2.1 Search strategy and selection criteria

This systematic review and meta-analysis is reported in accordance with the Preferred Reporting Items for Systematic Reviews and Meta-Analyses (PRISMA) Statement and was registered at the International Prospective Register of Systematic Reviews (number CRD42019124876).

To retrieve all the required studies, we searched PubMed/Medline, EMBASE, and the Cochrane Central Register of Controlled Trials from its inception to December 2019 to identify RCTs that compared the prognosis of short-term high-dose statin (a high-dose statin is defined as a dose of a statin that, on average, reduces low-density lipoprotein-cholesterol by ≥50% regardless of the type of statin, including atorvastatin 40–80 mg/d and rosuvastatin 20–40 mg/d) pretreatment with control (low-dose or no statin) in East Asian patients who were undergoing PCI. Search terms were Hydroxymethylglutaryl-CoA Reductase Inhibitors, HMG-CoA Reductase Inhibitors, Statins, HMG-CoA, rosuvastatin, atorvastatin, Fluvastatin, pitavastatin, pravastatin, simvastatin, Percutaneous Coronary Interventions, Percutaneous Coronary Revascularization, PCI, stents, angioplasty, randomized controlled trials, and RCT. References in the selected studies were also reviewed for additional eligible studies. There were no language limits in this study.

### 2.2 Study selection and data extraction

Based on the PICOS principle, we set the inclusion criteria as follows: (1) East Asian patients who were diagnosed with stable angina or acute coronary syndrome (ACS) underwent primary or elective PCI; (2) high-dose statin (all types) or control (low-does or no statin) patients were pretreated before the PCI; (3) data on rates of 30-day MACE (eg, all-cause death, target vessel revascularization, recurrent angina, or myocardial infarction, and sever heart failure) or PMI were reported; and (4) RCTs. The exclusion criteria were as follows: (1) non-RCTs or RCTs that were not well performed; (2) target events were underreported; (3) articles only had abstracts from conference proceedings; (4) the PMI or MACE rate was not described or could not be calculated; and (5) the study participants were non-East Asian. In the present study, 2 reviewers (Zhang and Liu) independently screened the articles for the eligibility criteria. Disagreements were resolved by consensus.

Two reviewers (Zhang and Liu) extracted the information from eligible studies independently, including the following information: the first author's name, publication year, and trial sample size; baseline characteristics of patients from each study, including age, sex, clinical classification (stable angina or ACS), type of population (statin naïve or not), type of statin, statin regimen before and after PCI, follow-up period, and comorbidity; and outcomes (incidence of PMI, MACE). The PMI definition was taken from the original articles. MACE included death, spontaneous myocardial infarction (MI), and target vessel revascularization at the longest follow-up for each study. Any disagreement was verified by both sides.

The methodological quality of the included studies was independently assessed by 2 reviewers (Zhang and Liu) using the Cochrane Collaboration and the Quality of Reporting of Meta-analyses (QUORUM) guidelines. Any disagreements were resolved by discussion.

### 2.3 Statistical analysis

In this meta-analysis, we used the RevMan 5.3 software (Copenhagen: The Nordic Cochrane Centre, The Cochrane Collaboration, 2014). The incidence of PMI and MACE, as the dichotomous outcomes, was evaluated using the odds ratios (ORs) and 95% confidence intervals (CIs). Heterogeneity across trials was evaluated using the *I*^2^ statistic, which is defined as *I*^2^ > 50%. If heterogeneity was present, a random-effects model was used; otherwise, a fixed-effects model was chosen. We also conducted a sensitivity analysis, in which 1 study at a time was removed and the others were analyzed to estimate whether the results could have been influenced markedly by a single study. Potential publication bias was assessed using funnel plots. All tests were two-tailed, and a *P* value of less than .05 was considered to be statistically significant. Study results were grouped by the following study characteristics: stable angina compared with ACS, and statin-naïve or a history of taking statins.

## 3 Results

**Figure 1:**
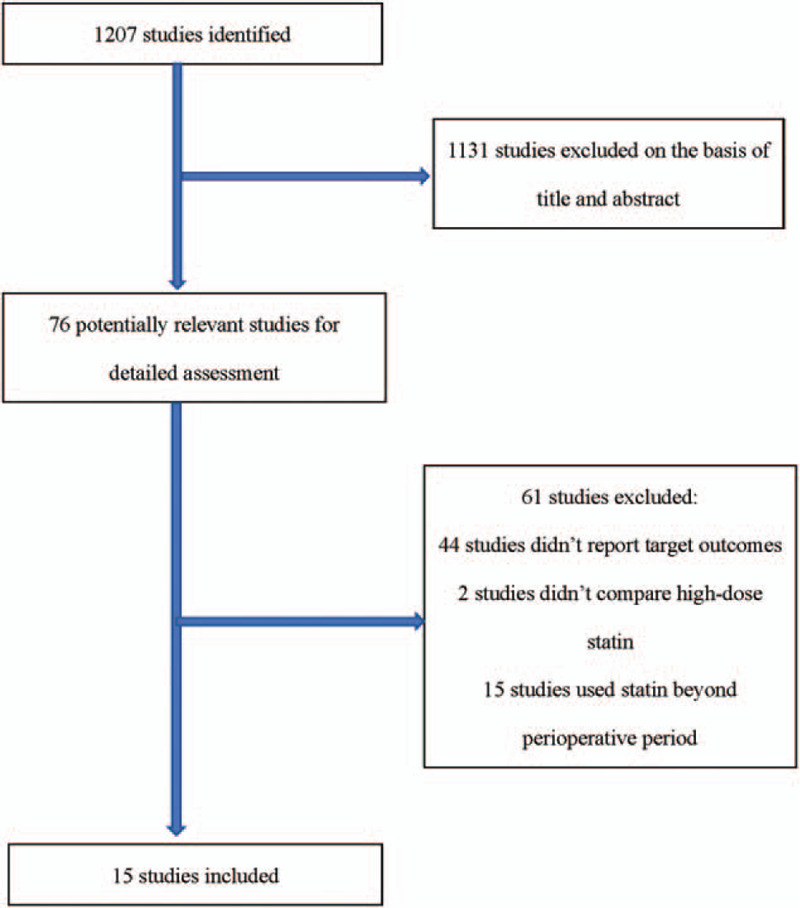
Study selection.

We identified 1207 potentially relevant publications from the electronic database, most of which were excluded because they had no relevance to our analysis, and the full text of 76 articles were reviewed. Fifteen studies^[6–20]^ were included in this analysis (Fig. 1): 12 studies included patients who were completely statin-naïve (without a history of administering any kind of statins); eight studies included patients who were non-ST-segment elevation ACS (NSTE-ACS), 2 studies enrolled patients with ST-segment elevation myocardial infarction (STEMI); 6 studies included patients who had stable CAD; 6 studies enrolled patients who received pretreatment with rosuvastatin; 10 studies enrolled patients who received atorvastatin before the PCI; 10 studies reported the incidence of PMI; and 13 studies reported the rates of MACE. All trials were published between 2009 and 2016.

This meta-analysis included 4313 patients, among whom 2128 (49.3%) patients were in the high-dose statin pretreatment group and 2185 (50.7%) patients were in the control group. Characteristics of the included studies are presented in Table 1. There were 69.5% and 71.1% male patients in the control and statin groups, respectively. There was no significant difference in comorbidities (ie, hypertension, diabetes mellitus, and hyperlipidemia) between the 2 groups (details of baseline patient characteristics and procedural data from included studies were listed in Table 2). Most of the included patients were statin-naïve, and 11 of 15 studies were conducted in China.

**Table 1:** Characteristics of studies included in the meta-analysis.

Trial	Patients (n)	Nation	Type of population	Clinical presentation	Type of statin	Statin regime before PCI	Statin regime after PCI	Follow up	Definition of PMI	Definition of MACE
Yun et al 2009	445	Korea	Statin-naïve	NSTE-ACS	Rosuvastatin	Rosuvastatin 40 mg loading for 16 ± 5 h before PCI VS no statin	NA	1 month	CK-MB >2UNL	Death, Q wave MI, TVR, ischemic stroke
Kim et al 2010	171	Korea	Statin-naïve	STEMI	Atorvastatin	80 mg atorvastatin before PCI VS 10 mg atorvastatin	10 mg qn atorvastatin	9 months	CK-MB >3UNL	Death, nonfatal MI (including PMI), and TVR
Tang et al 2010	103	China	Statin-naïve	CHD	Atorvastatin	40 mg atorvastatin 1-3 d before PCI VS no statin or 20 mg atorvastatin	20 mg qn atorvastatin	NA	cTNI>3UNL	/
Yu et al 2011	81	China	Statin-naïve	NSTE-ACS	Atorvastatin	80 mg loading dose given 12 h with a further 40 mg given 2 h before PCI VS placebo	20 mg qn atorvastatin	1 month	CK-MB >2UNL	Cardiac death, nonfatal AMI, or TVR
Li et al 2013	215	China	Long-time statin therapy	Stable angina	Atorvastatin	80 mg atorvastatin 12 h before PCI VS 20 mg atorvastatin	20 mg qn atorvastatin	1 month	/	Relapse angina, MI, cardiac death, and stent thrombosis or TVR
Luo et al 2013	67	China	Statin-naïve	NSTE-ACS	Rosuvastatin	20 mg rosuvastatin 12 h and a further 20 mg 2 h preprocedure dose VS no statin	NA	1 month	cTNI>3UNL	MI, death, ischemic stroke, and stent thrombosis or revascularization
Takano et al 2013	210	Japan	37.6% were statin-naïve	Stable CAD	Rosuvastatin	20 mg rosuvastatin 5 to 7 days before PCI VS 2.5 mg rosuvastatin each day	Long-term rosuvastatin	1 year	CK-MB >3UNL	/
Wang et al 2013	125	China	Statin-naïve	NSTE-ACS	Rosuvastatin	20 mg rosuvastatin 2–4 h before PCI VS placebo	10 mg qn rosuvastatin	1 month	CK-MB >3UNL	Cardiac death, MI, and TVR
Jang et al 2014	335	Korea and China	Statin-naïve	NSTE-ACS	Atorvastatin	80 mg atorvastatin 12 h and 40 mg 2 h pre-PCI VS no statin	40 mg qn atorvastatin	1 month	/	Death, MI, and TVR
Xie et al 2014	159	China	Statin-naïve	NSTE-ACS	Rosuvastatin	20 mg rosuvastatin 12 h and a further 20 mg 2 h preprocedure dose VS placebo	NA	NA	cTN>5UNL	Death of cardiac or procedure- related origin, MI, and repeat TVR, ischemic stroke and stent thrombosis
Guo et al 2015	112	China	Statin-naïve	CHD patients aged over 80 years underwent elective PCI	Atorvastatin	40/60 mg atorvastatin within 48–72 h pre-PCI VS 20 mg atorvastatin	40 mg qn atorvastatin	1 month	cTN>5UNL	Cardiac death, MI, and TVR
Jiao et al 2015	72	China	NA	NSTE-ACS with diabetes	Rosuvastatin	20 mg rosuvastatin 12 h and a further 20 mg just before PCI VS standord method	10 mg qn rosuvastatin	1 month	/	Death, MI, angina pectoris, HF, TVR
Jo et al 2015	218	Korea	Statin-naïve	STEMI	Atorvastatin	80 mg atorvastatin before PCI and 80 mg/d for 5 days after the PCI VS 10 mg qn atorvastatin	10 mg qn atorvastatin	6 months	/	Death, MI, HF, TVR
Zheng et al 2015	1202	China	Most of the participants were statin-naïve	NSTE-ACS	Atorvastatin	Atorvastatin 80 mg/day for 2 days before CAG VS standord method	atorvastatin 40 mg/day for 30 days post-PCI and then treated with usual care	6 months	/	Cardiac death, MI, or TVR
Liu et al 2016	242	China	Statin-naïve	Stable angina	Atorvastatin	80 mg 12 hours before PCI and 40 mg/d thereafter VS no statin	20 mg qn atorvastatin	1-year	CK-MB >3UNL.	Cardiovascular death, spontaneous MI, and unplanned revascularization
	556			ACS						

AMI = acute myocardial infarction, CAD = coronary artery disease, CHD = coronary heart disease, CK-MB = creatine kinase MB, cTNI = cardiac troponin I, HF = heart failure, MACE = major adverse cardiac events, NSTE-ACS = non-ST-segment elevation ACS, PCI = percutaneous coronary intervention, PMI = periprocedural myocardial infarction, STEMI = ST-segment elevation myocardial infarction, TVR = target vessel revascularization, ULN = upper limit of normal.

**Table 2:** Baseline patient characteristics and procedural data.

	Age		Male		BMI		Current smoking		Hypertension		Diabetes		Dyslipidemia		Total stent length		Multivessel lesion	
Trial	C	S	C	S	C	S	C	S	C	S	C	S	C	S	C	S	C	S
Yun 2011	63 ± 11	64 ± 10	137	136	/	/	80	83	121	123	65	75	3.2 ± 1.03	3.15 ± 0.98	46 ± 29	45 ± 24	118	126
Kim 2010	59 ± 11	61 ± 11	66	66	/	/	43	35	39	45	16	21	32	34	31.3 ± 11.9	30.6 ± 11.3	45	59
Tang 2010	64.2 ± 8.92	66.08 ± 9.79(49%); 60.16 ± 8.65(51%)	22	53	23.3 ± 3.06	24.88 ± 2.91(49%); 24.33 ± 3.03(51%)	7	31	15	50	4	14	7	10	/	/	/	/
Yu 2010	64.4 ± 11.6	63.3 ± 10.5	23	25	/	/	16	18	18	20	15	14	19	20	16.7 ± 5.3	16.8 ± 5.4	35	35
Li 2013	58.4 ± 9.5	60.2 ± 11.1	66	67	/	/	57	53	73	58	67	64	2.6 ± 1.5	2.6 ± 1.2	29 ± 10	30 ± 9	/	/
Luo 2013	61 ± 9	58 ± 12	28	28	/	/	24	19	23	18	10	10	2.9 ± 0.8	2.8 ± 1.1	30 ± 10	28 ± 10	28	17
Takano 2013	68 ± 9	69 ± 10	81	81	/	/	31	26	76	80	55	54	2.92 ± 0.73	2.9 ± 0.67	/	/	/	/
Wang 2013	64.8 ± 11.6	65.4 ± 10.2	41	40	25.2 ± 2.9	24.6 ± 2.3	23	21	47	45	19	20	2.4 ± 0.9	2.5 ± 0.9	35.8 ± 18.3	32.6 ± 16.8	23	20
Jang 2014	60 ± 10	61 ± 9.2	126	116	/	/	/	/	108	95	38	32	37	27	/	/	/	/
Xie 2014	59.8 ± 10.5	61.5 ± 11.4	56	59	24.9 ± 3.2	25.5 ± 3.4	45	40	45	41	21	19	2.7 ± 1.1	2.9 ± 0.9	29 ± 11	30 ± 10	60	56
Guo 2015	81.82 ± 1.35	82.325 ± 3.115	18	40	/	/	6	13	21	54	6	16	2.93 ± 1.06	3.015 ± 0.83	29.5 ± 13.6	32.05 ± 13.94	9	19
Jiao 2015	60.9 ± 10.7	59.3 ± 11.8	26	21	/	/	12	9	15	13	39	33	3.22 ± 0.75	3.48 ± 0.81	17.9 ± 5.3	18.6 ± 5.7	30	26
Jo 2015	61 ± 12.3	57.6 ± 13	90	95	24.1 ± 3	24.6 ± 3	52	67	47	47	25	33	53	64	/	/	/	/
zheng 2015	59.7 ± 8.36	59.47 ± 8.6	455	421	24.99 ± 3.12	25.15 ± 3.11	222	214	416	366	165	136	147	125	48.65 ± 30.15	48.36 ± 30	449	420
Liu 2016	62.5 ± 11.2	61.8 ± 10.1	284	293	27.1 ± 4.8	27.3 ± 4	79	86	255	260	130	127	3.4 ± 1.1	3.4 ± 0.9	32.4 ± 13.7	31.2 ± 12.3	106	112

BMI = body mass index, C = control group, S = statin group.

**Figure 2:**
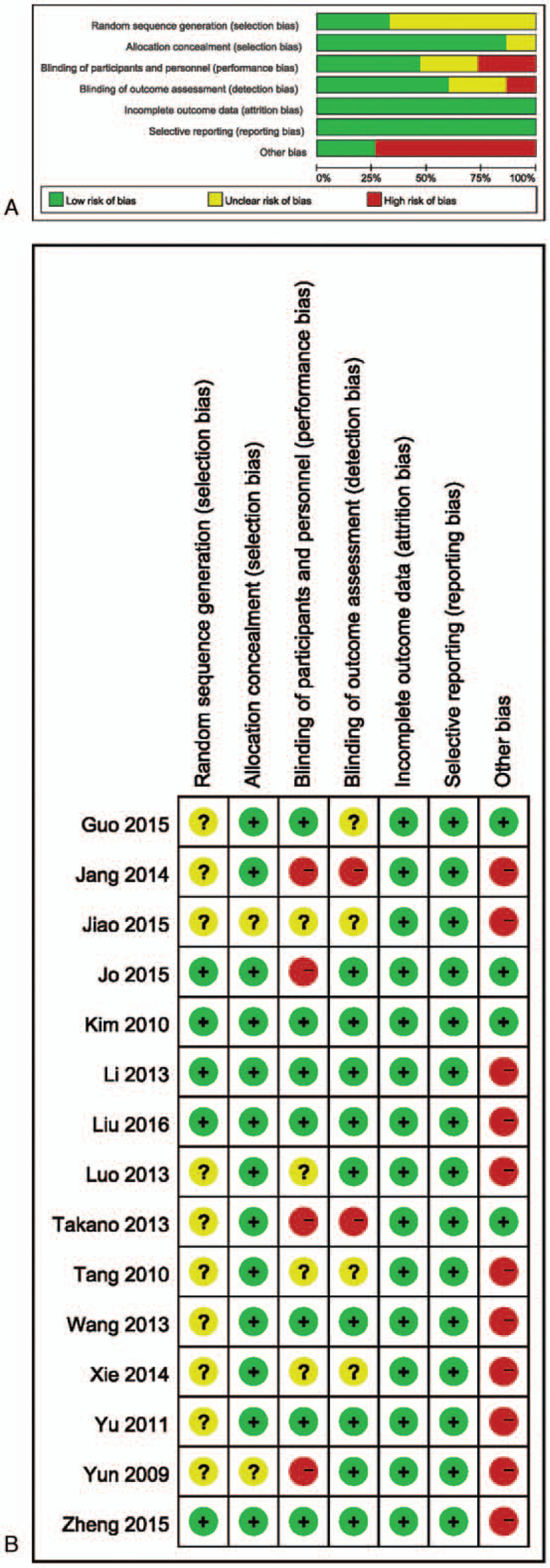
(A) Risk of bias graph. (B) Risk of bias summary.

The risk of bias assessment is shown in Fig. 2. All 15 RCTs reported complete outcomes and results, no trial was stopped early. However, 8 studies that included NSTE-ACS patients were rated as high risk of bias because of patients with non-specific symptoms such as chest pain, in whom it was difficult to determine if the patient had unstable angina.

**Figure 3:**
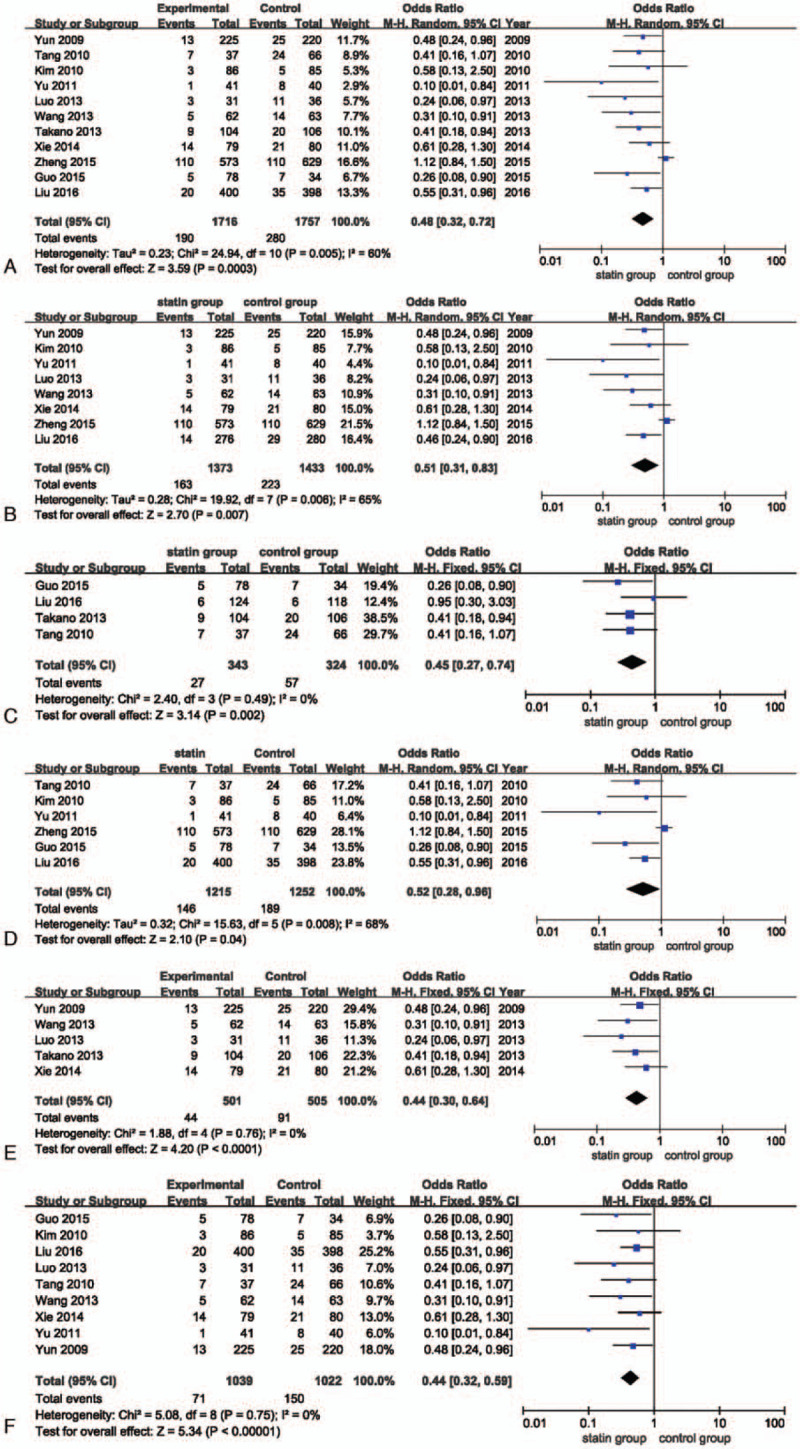
(A) Forest plot of the PMI incidence. (B) Forest plot of the PMI incidence in the ACS group. (C) Forest plot of the PMI incidence in the stable CHD group. (D) Forest plot of the PMI incidence in the atorvastatin group. (E) Forest plot of the PMI incidence in the rosuvastatin group. (F) Forest plot of the PMI incidence in the statin-naive group. ACS = acute coronary syndrome, CHD = coronary heart disease, PMI = periprocedural myocardial infarction.

In a pooled analysis of all the trials, compared with patients who received low or no statins before the PCI, patients who received a high pretreatment dose of statins had a statistically significant reduction (52%) in the risk of PMI (OR, 0.48; 95% CI, 0.32–0.72; *P* < .001; *I*^2^, 60%; Fig. 3A). Similarly, despite patients who presented with ACS (including NSTE-ACS and STEMI) or stable CHD, the incidence of PMI in the high-dose statin pretreatment group showed a greater reduction compared with the control group (OR, 0.51; 95% CI, 0.31–0.83; *P* = .007; *I*^2^, 65% vs OR, 0.45; 95% CI, 0.27–0.74; *P* = .002; *I*^2^, 0%; Fig. 3B and C). When taking statin types into consideration, both high-dose atorvastatin and rosuvastatin pretreatment groups were associated with lower rates of PMI (OR, 0.52; 95% CI, 0.28–0.96; *P* = .04; *I*^2^, 68% vs OR, 0.44; 95% CI, 0.30–0.64; *P* < .001; *I*^2^, 0%; Fig. 3D and E). Among statin naïve patients, the high-dose statin pretreatment group also showed a relative reduction in the PMI rate compared with the control group (OR, 0.44; 95% CI, 0.32–0.59; *P* < .001; *I*^2^, 0%; Fig. 3F).

**Figure 4:**
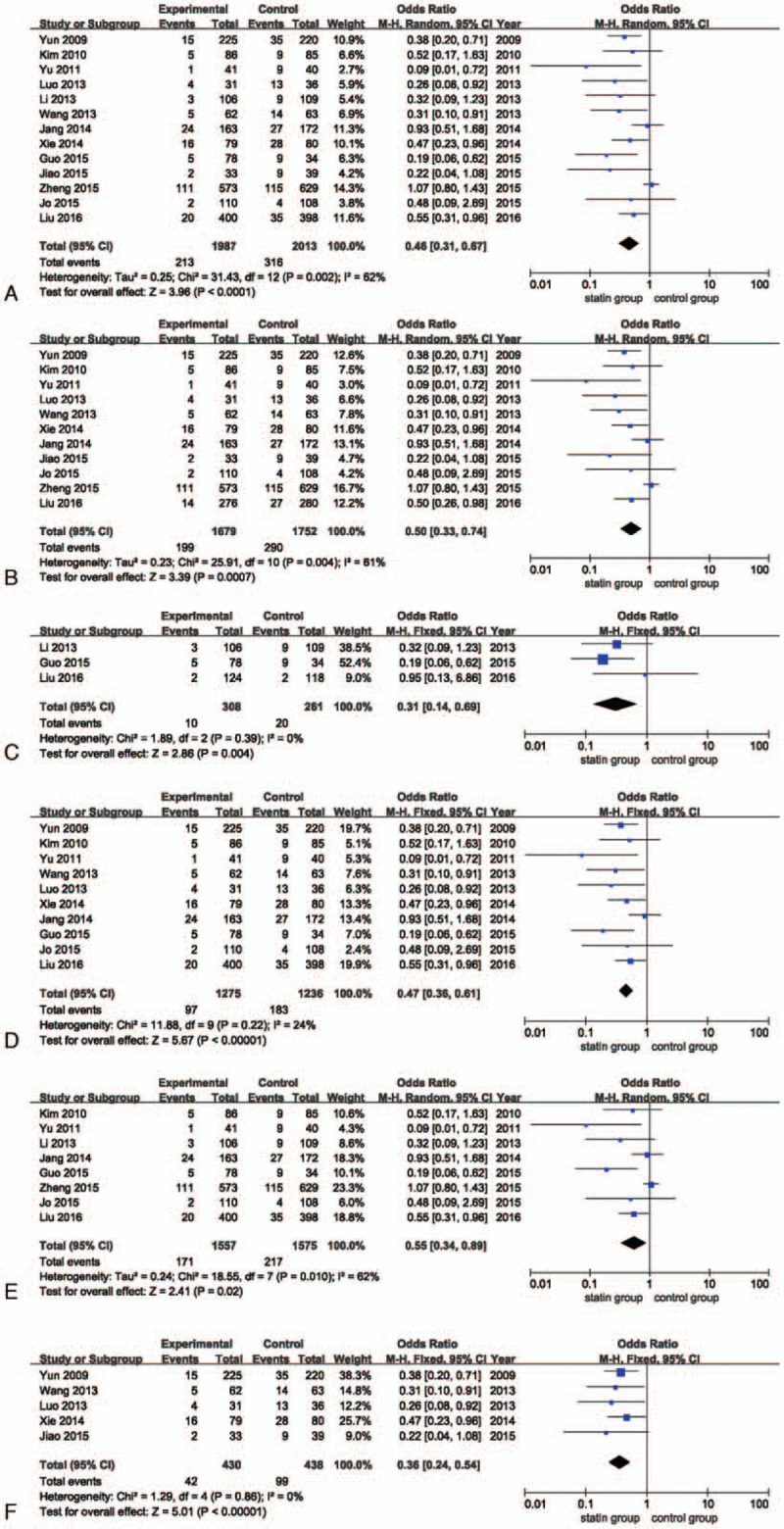
(A) Forest plot of the MACE incidence. (B) Forest plot of the MACE incidence in ACS patients. (C) Forest plot of the MACE incidence in stable CHD patients. (D) Forest plot of the MACE incidence in statin-naive patients. (E) Forest plot of the MACE incidence in the atorvastatin group. (F) Forest plot of the MACE incidence in the rosuvastatin group. ACS = acute coronary syndrome, CHD = coronary heart disease, MACE = major adverse cardiac events, PMI = periprocedural myocardial infarction.

The overall outcome data for MACE were available from 13 RCTs, which were analyzed using the random-effects model, and these data indicate that high-dose statin pretreatment led to a 54% relative reduction in MACE (OR, 0.46; 95% CI, 0.31–0.67; *P* < .001, *I*^2^, 62%; Fig. 4A). To further confirm whether the outcomes of high-dose statin pretreatment before PCI differed from patients who presented with stable angina, or ACS, we assessed subgroups of patients based on their clinical type. High-dose statin pretreatment was associated with a 50% relative reduction in MACE for patients with ACS (OR, 0.50; 95% CI, 0.33–0.74; *P* < .001, *I*^2^, 61%; Fig. 4B), and a 69% relative reduction in stable angina (OR, 0.31; 95% CI, 0.14–0.69; *P* = .004, *I*^2^, 0%; Fig. 4C).

We also explored the effect of the history of statins on MACE. For patients who had no history of taking statins, the incidence of MACE was lower in the high-dose statin group compared with the control group (OR, 0.47; 95% CI, 0.36–0.61; *P* < .001, *I*^2^, 24%; Fig. 4D). Eight studies that enrolled atorvastatin patients showed that high-dose statin pretreatment caused a 45% reduction in the high-dose statin group compared with the control group (OR, 0.55; 95% CI, 0.34–0.89; *P* = .02, *I*^2^, 62%; Fig. 4E). Additionally, other studies with rosuvastatin pretreatment showed that high-dose statin pretreatment caused a 64% reduction in the high-dose statin group compared with the control group (OR, 0.36; 95% CI, 0.24–0.54; *P* < .001, *I*^2^, 0%; Fig. 4F).

### 3.1 Adverse events

Among the included studies, there were no reports of rhabdomyolysis or hepatic failure in the high-dose statin group.

### 3.2 Sensitivity analysis

We investigated the effect of a single study on the overall risk estimate by excluding 1 study at a time. The combined OR of overall risk estimates were consistent and without apparent fluctuation, with a PMI incidence of 0.30 (95% CI, 0.18–1.52) to 0.49 (95% CI, 0.32–0.75) and a MACE incidence of 0.41 (95% CI, 0.27–0.63) to 0.49 (95% CI, 0.34–0.72).

## 4 Discussion

This systematic review and meta-analysis evaluated the effect of high-dose atorvastatin pretreatment on the PMI and MACE incidence in the East Asian population undergoing either emergency PCI or elective PCI. The main finding is that high-dose statin therapy can lead to a significant benefit for 30-day MACE and periprocedural MI in East Asian patients undergoing PCI.

PCI has become a widely used and effective therapy for ischemic heart disease, and technical and pharmacologic advances have reduced the occurrence of severe complications. However, PMI remains a common phenomenon and a main component of MACE. It was reported that 23% of patients have increased creatine kinase MB (CK-MB) above the upper limit of normal (ULN) after PCI, with most elevations being 1 to 3-times the ULN, and 27% of patients had an increase in troponin I levels.^[21]^ CK-MB increased to greater than 3 times the ULN in 6% to 18% of PCIs.^[22,23]^ The degree of CK-MB elevation independently predicted the risk of death and there was a dose-response relationship between the degree of CK-MB increase and the risk of death.^[24–27]^ The PCI guidelines published by the American College of Cardiology (ACC) in 2011 provide a class IIb recommendation for routine CK-MB and troponin measurement after PCI in all patients regardless of their symptoms to allow prognostic evaluation.^[28]^ Previous studies conducted in Caucasian populations have suggested that patients undergoing PCI can benefit from periprocedural high statin loading use. In the randomized ARMYDA trial,^[29]^ 7-day pretreatment with atorvastatin (40 mg/d) before PCI in patients with stable angina was associated with an 80% risk reduction for PMI. In the ARMYDA-ACS trial,^[30]^ the incidence of PMI was reduced during PCI in high-loading atorvastatin pretreatment with ACS. The ARMYDA-RECAPTURE trial^[31]^ showed that short-term pre-treatment with a high-dose atorvastatin load before PCI improves the outcome in patients who are already receiving chronic statin therapy. Similarly, the SECURE-PCI^[32]^ revealed that among 2710 ACS patients (both statin-naïve patients and patients with a history of taking statins) who underwent PCI, 30-day MACE occurred in 81 (6.0%) of 1351 patients in the atorvastatin group compared with 112 (8.2%) of 1359 in the placebo group (HR, 0.72; 95% CI, 0.54–0.96; *P* = .02). However, the ALPACS study^[13]^ that was conducted in statin-naïve Korean and Chinese patients with NSTE-ACS who received additional atorvastatin loading pre-PCI, did not find a beneficial effect compared with usual atorvastatin treatment. Another study, the ISCAP trial,^[19]^ demonstrated that serial intensive atorvastatin therapy did not improve the clinical outcome among Chinese patients undergoing elective PCI. The differences in baseline characteristics, coronary lesions, and procedures among the studies could affect the outcomes. Most PMI events were related to the amount of atherosclerotic plaque burden, side branch flow impairment, or occlusion during bifurcation intervention.^[26]^ Thus, patients with multivessel disease, multiple or long lesions, or diffusely diseased arteries had a larger atherosclerotic burden and were more prone to PMI. In addition, stent length, stent overlap, and the Taxus design are associated with an increased risk of branch vessel compromise, which is a risk factor for PMI.^[21]^ In the ARMYDA-ACS trial, interventions included 19% and 22% multivessel interventions in the atorvastatin intensive group and the control group, respectively. In accordance with the ISCAP trial, the multivessel intervention incident rates were 32.3% and 34.2% in the atorvastatin intensive and control groups, respectively. The ISCAP trial also used a longer total stent length and more stents compared with previous studies. In ARMYDA-ACS, the PMI rate in the placebo arm was 15%, which was lower compared with the ISCAP and ALPACS trials. Patients with complex coronary lesions and a subsequently complicated intervention may attenuate the effect of high-dose statin treatment on reducing the PMI. Other possible confounding variables, such as the ACS diagnostic category, use of statins in the control groups, statin administration time, and procedure-related complications may all affect the PMI occurrence.

Kim et al^[6]^ and Jo et al^[18]^ each conducted studies where 80 mg atorvastatin loading pretreatment in patients with STEMI who were undergoing primary angioplasty. Both studies did not show positive results. Because of the door-to-balloon time requirement in STEMI, the short action time of atorvastatin may be an important explanation for these results. In addition, the ischemic burden or injury might be too severe to be prevented or reduced by a single dose of atorvastatin before primary PCI. Total obstruction of epicardial coronary arteries was found more frequently, and the infarct size was greater in STEMI compared with NSTE-ACS.^[33]^ Post-procedural MI is difficult to distinguish from the original MI in practice. Although PMI occurrence showed no reduction compared with the control group, Kim et al study^[6]^ suggested that high-dose atorvastatin loading before PCI may improve microvascular coronary perfusion as determined by CTFC, MBG, and STR after PCI. Moreover, in the SECURE-PCI study,^[32]^ in the subgroup of patients with STEMI who were undergoing primary PCI, 80 mg of atorvastatin that was administered before the treatment showed a significant reduction in MACE at 30 days.

Currently available evidence from RCTs and meta-analyses suggests that the clinical benefit of statin treatment is largely driven by the absolute LDL-C reduction. For each 1-mmol/L reduction in LDL-C, statins reduced major vascular events (MI, CAD death, or any stroke or coronary revascularization) by 22%, major coronary events by 23%, CAD death by 20%, total stroke by 17%, and total mortality by 10% over 5 years.^[34]^ Thus, it is recommended that a high-intensity statin is prescribed up to the highest tolerated dose to reach the goals set for the specific level of risk.^[35–37]^ Short-term pretreatment with high-dose statins did not have a significant effect on cholesterol levels, and the possible mechanism underlying the early protective effect of statins by non-lipid-related mechanisms is called a pleiotropic effect. Many studies demonstrated that a single high dose of statin promptly modifies inflammatory responses, plaque stability, and thrombus formation.^[38]^ The guideline recommends that routine pre-treatment or loading (on the background of chronic therapy) with a high-dose statin should be considered in patients undergoing PCI for an ACS or elective PCI (IIa, B).^[37]^

The population in East Asia may have better statin responsiveness and lower baseline LDL-C compared with people from North America and Europe. There is an ethnic difference in the statin pharmacokinetics in Asian people compared with western people, which is partly related to pharmacogenetic differences. Chinese people have a higher frequency of the ATP binding cassette G2 c.421A variant compared with Caucasian people, and this contributes to the higher plasma concentrations of rosuvastatin and greater reductions in LDL-C in East Asian individuals.^[39–41]^ Therefore, 20 mg daily of rosuvastatin in East Asian patients would result in the same average systemic exposure as the maximum approved dose of 40 mg in Caucasian patients.^[42,43]^ The apparent increased statin response is probably partly related to lower baseline levels of LDL-C in East Asian people compared with Caucasian people so that more patients can reach the LDL-C goal with a certain statin dose. In Japan, the target LDL-C level for secondary prevention was previously less than 100 mg/dL.^[44]^ However, the 2017 Japan Atherosclerosis Society Guidelines specify the target goal in patients with ACS to be 70 mg/dL.^[34,45]^ The REAL-CAD study that enrolled 13,054 participants in Japan showed that high-dose pitavastatin (4 mg/d) significantly reduced MACEs.^[46]^ Based on the studies, the 2018 Japanese Circulation Society (JSC) guideline on the diagnosis and treatment of ACS recommends that the maximum tolerable dose of a strong statin should be administered in ACS Japanese patients (I,A).^[47]^ However, the guideline in China still doubts the safety of high-dose statins.^[48]^ Our current meta-analysis shows that high-dose statin pretreatment can result in a significant reduction in 30-day MACE and PMI for Asian patients undergoing PCI. Among the included studies, the risk of a safety outcome with high-dose statins is rare and comparable to the usual statin dose. Thus, these findings may provide practical information in these clinical scenarios.

There is, in addition, 1 further point to claim. Among a variety of risk factors contributed to CAD, smoking and dyslipidemia are 2 most important risk factors, accounting for 2/3 of all risk factors.^[49]^ Statins proved to be able to prevent nicotine effects by modulating activation of Rho A and NF-κB pathways.^[50]^ The studies included in this meta-analysis report different proportions of smokers. Lacking specific data, further clinical trials need to be conducted to clarify this interaction between smoking and the effect of statin.

## 5 Limitations

There are several limitations to this meta-analysis. First, because the definition of PMI has changed over time, the diagnostic definitions of PMI were varied among included studies, as well as the time points at which cardiac markers were measured after PCI. Moreover, we had no access to patient-level data, so details of each study procedure complication were lacking. It has been acknowledged that side-branch occlusion, flow-limiting dissection, and abrupt vessel closure might all lead to perioperative MI. Third, antithrombotic treatments used in the various trials appear to be different, and the clinical benefit of high-dose statins that was observed in the present meta-analysis may be affected. Finally, Of 15 RCTs included in this analysis, the latest study was publicated in 2016.

## 6 Conclusion

High-dose statin pretreatment can result in a significant reduction in 30-day MACE and PMI for East Asian patients undergoing PCI, regardless of the clinical presentation and statin type.

## Acknowledgments

We thank all the investigators who participated in this meta-analysis. We also thank Jodi Smith, PhD, from Liwen Bianji, Edanz Editing China (www.liwenbianji.cn/ac), for editing the English text of a draft of this manuscript.

## Author contributions

**Conceptualization:** Jiahui Liu, Bin Zhang, Ming Chen, Bo Zheng.

**Data curation:** Jiahui Liu, Bin Zhang.

**Formal analysis:** Jiahui Liu.

**Methodology:** Jiahui Liu, Bin Zhang.

**Software:** Jiahui Liu.

**Supervision:** Ming Chen, Bo Zheng.

**Validation:** Bo Zheng.

**Writing – original draft:** Jiahui Liu.

**Writing – review & editing:** Jiahui Liu.
